# A topological model of biofeedback based on catecholamine interactions

**DOI:** 10.1186/1742-4682-2-11

**Published:** 2005-03-21

**Authors:** Tapas K Basak, Suman Halder, Madona Kumar, Renu Sharma, Bijoylaxmi Midya

**Affiliations:** 1 Department of Electrical Engineering, Jadavpur University, Kolkata-700032, India

**Keywords:** Biofeedback, Transduction Phase, Catecholamine, Psychosomatic Disease, Activation of smooth muscles

## Abstract

**Background:**

The present paper describes a topological model of biofeedback. This model incorporates input from a sensory organ and a transduction phase mediated through catecholamine production in the feedback path. The transduction phase comprises both conservative and dissipative systems, from which the appropriate output is combined in a closed loop.

**Results:**

The model has been simulated in MATLAB 6.0 R12 in order to facilitate a comprehensive understanding of the complex biofeedback phenomena concomitant with the transduction phases associated with migraine and with psychosomatic diseases involving digestive disorders.

**Conclusion:**

The complexity of the biological system influences the transduction phase and nature of the system response, which is consequent on the activation of smooth muscles by sympathetic and parasympathetic stimulation.

## Background

The paper describes a comprehensive model of a biofeedback system; it adopts a new approach to modeling. Using artificial neural networks (ANN) it is not easy to obtain a dynamic response that reflects dependence on hormone production. Therefore, the authors have endeavoured to design an approach that focuses on the internal state of the subject consequent on biofeedback stimulation.

**Figure 1:**
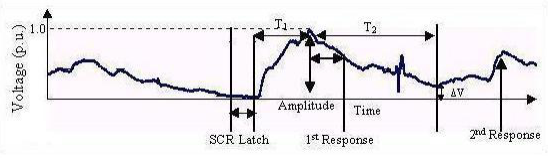
Generalized Galvanic Skin Response.

A biofeedback system involves a sensory organ and an appropriate stimulus. The stimulus is mediated through organs derived from specific biosensors [2-8]. If a subject has disorders involving parenchymal lesions, his or her internal state is likely to indicate exhaustion, as evident from output responses in a conservative system (see below). Thus, it is or may be possible to establish the internal state of the subject from the output responses. The model described in this paper has been developed primarily with a focus on the galvanic skin response (GSR) in biofeedback [9]; galvanic skin response training is also known as the electrodermal response (EDR). The device measures electrical conductance in the skin, which is associated with the activity of the sweat glands [9,10]. Sweat gland activity is due to catecholamine secretion resulting from the stimulation of adrenergic receptors (discussed later). The GSR in a biofeedback system is caused by a stimulus that activates the sweat glands. This activation can be indicated by recording bio-potentials by placing the electrodes on the body surface. The instrumentation for recording consists of a set of amplifiers and filters designed for the purpose [9,10] (Fig. 1).

If T_1 _is the duration of the rising phase, T_2 _is the duration of the decaying phase and ΔV is the residual homeostatic output level, the result from Fig. 1 is tabulated below (Table 1).

**Table 1:** Records of the measurements of the SCR

Measure	Measured value	Measure	Measured value	Per unit value
SCR latency	3s	Peak response	84.5 mV	1 p.u.
SCR rise time	9.69s	Amplitude	25.5mV	0.3 p.u.
Half recovery time	8.75s			

Before we focus on the design of the biofeedback system, some important terminology needs to be discussed: *Topological model, Transduction Phase, Unity biofeedback, Homeostasis, Homeostat, Residual Homeostatic output level, Feedback Control systems, Catecholamine Interactions, Conscious and subconscious parts of the brain and Dissipative & Conservative system*.

A *topological model *originates from a root and spreads in tree-like branches. It affords a complete description of the interactions among the different parts of the system considered. The *transduction phase *of a subject reflects physiological changes caused by hormone release consequent on stimulation. This phase is characteristic of an individual subject [2-7]. For example, the transduction phase of a psychosomatic patient is sometimes reflected during a journey in a high-speed vehicle, when the physiological outcome can adversely affect his mental condition, associated with headache and vomiting.

*Unity biofeedback *means that the homeostatic output is directly fed to the brain without going through the transduction phase, which incorporates conservative and dissipative systems. *Homeostasis *is the set of processes by which constant or 'static' conditions are maintained within the internal environment of a subject [6,7,11]; a *homeostat *is a controller involved in maintaining homeostasis.

In this paper the *residual homeostatic output level*, ΔV, has a particular value for each successive response. It can be correlated with the GSR [9]. The residual homeostatic output arises as a result of sustained catecholamine action, which often persists for minutes or hours; control is prolonged, not just instantaneous activation or inhibition [11]. The residual homeostatic output indicated by the GSR response signifies that sweating persists even after the withdrawal of the biofeedback stimulus [9].

Mammals are endowed with a vast network of *feedback control systems *with controllers (homeostats) without which survival would be difficult [11]. In this control system a particular neuro-hormone exerts a negative feedback effect, preventing over-secretion of other hormones associated with over-activity of the muscles, unless there is specific disorder in the system [11].

*Catecholamine interactions *are very important in biofeedback systems. Catecholamines are excitatory or inhibitory neurotransmitters or hormonal agents. The catecholamine neuro-hormones are *epinephrine, norepinephrine, dopamine and serotonin*. Epinephrine and norepinephrine function as excitatory hormones. Serotonin functions as an inhibitory hormone, and dopamine is excitatory in some areas and inhibitory in others. Stimulation of sympathetic nerves in the adrenal medullae causes large quantities of epinephrine and norepinephrine to be released into the circulating blood, which carries them to all tissues of the body. Norepinephrine increases the total peripheral resistance and thus elevates the arterial pressure; epinephrine raises the arterial pressure to lesser extent but increases the cardiac output more. Epinephrine has a 5 to 10 times greater metabolic effect than norepinephrine [11].

The adrenergic receptors include α and β receptors. The α-receptors control such physiological activities as vasoconstriction, iris dilatation, intestinal relaxation, intestinal sphincter contraction, pilomotor contraction and bladder sphincter contraction; β-receptors control (e.g.) vasodilatation, cardio-acceleration, increased myocardial strength, intestinal relaxation, uterus relaxation, bronchodilatation, calorigenesis, glycogenesis, lipolysis and bladder wall relaxation. It is therefore evident that both α and β receptors have inhibitory and excitatory functions [11]. Blood pressure transduction phases are associated with activation of α and β receptors [4-6].

The cerebral cortex, which includes the *conscious *part of the brain, never functions alone but always in association with lower centres of the nervous system. In fact, the lower brain centres (or *subconscious *part of the brain) initiate wakefulness in the cerebral cortex [11]. The subconscious part of the brain performs vegetative functions; notably, the hypothalamus controls sympathetic and parasympathetic stimulation [11]. The sweat glands secrete large quantities of sweat when the sympathetic nerves are stimulated; they are controlled primarily by centers in hypothalamus that are usually considered to be parasympathetic centers [11]. Therefore, sweating could be called a parasympathetic function, although it is controlled by nerve fibres that are anatomically distributed through the sympathetic nervous system [11]. The sympathetic innervation of sweat glands results from a developmental change in transmitter phenotype (from catecholaminergic to cholinergic), making parasympathetic stimulation also possible [13].

In biofeedback systems, the subject undergoes different transduction phases. Depending on the nature of transduction phase a system can be classified as *dissipative *or *conservative*. A dissipative system diverges from its original state during biofeedback; it may undergo successive stages during which the response decreases exponentially, with the characteristic features of a normal physiological system. A conservative system, in contrast, has an output characterised by exponentially rising phases due to sustained levels of catecholamines.

Nowadays, biofeedback has important clinical applications in at least the following areas. Headache is a psychophysiological disorder associated with disturbances in the homeostatic relationship between mind and body. The classical psychosomatic disorders are included in this category, e.g. peptic ulcer, bronchial asthma, migraine and essential hypertension.[12] In classical migraine (in which the sufferer is sensitive to light and sound stimuli) there are neurological symptoms such as homonymous hemianopia, paresthesias, aphasia and hemiparesis, which precede the unilateral headache (tension headache) and are reflected in the subject's muscle activity [12]. Biofeedback is useful for migraine treatment. Stimulation or inhibition of specific adrenergic receptors, mediated through catecholamines, often help relieve the pain, inducing a feeling of drowsiness by a process associated with the smelling of ripe mango or fresh lemon [4].

The digestive system as a whole is governed by innumerable control mechanisms at the cell and tissue levels, whereby a pathway can be activated as needed or inhibited as products accumulate [12]. For example, acetylcholine is an excitatory cholinergic transmitter for smooth muscle fibers in some organs, but an inhibitory transmitter for smooth muscle in others. When acetylcholine excites a muscle fiber, norepinephrine ordinarily inhibits it. Conversely, when acetylcholine inhibits a fiber, norepinephrine usually excites it [11]. Cholinergic (muscarinic) receptors are involved in the parasympathetic activity. Muscarinic receptors are age dependent; their frequency decreases with increasing age. Moreover, the fall of blood pressure and pulse rate during parasympathetic stimulation (discussed later) is due to the combined effects of adrenergic and muscarinic receptors [14].

Adrenergic and cholinergic receptors in the autonomic nervous system play opposite roles. De-activation of the sympathetic innervation (which operates via adrenergic receptors) is followed by enhancement of the cholinergic receptors involved in parasympathetic stimulation in smooth muscle. Conversely, noradrenergic enhancement is diminished as cholinergic neurotransmission becomes established [14].

In the model discussed in this paper, the stimulation of adrenergic receptors diminishes concomitantly with blood pressure and pulse-rate (a dissipative system). This diminishing of the adrenergic receptor effect enhances cholinergic receptor activity automatically in the control of smooth muscle function. Similarly, in a conservative system, adrenergic receptor stimulation is enhanced concomitantly with the blood pressure and the pulse rate. This increasing effect of the adrenergic receptors will diminish the effects of cholinergic receptors automatically in the control of smooth muscle activity. Thus, cholinergic receptors automatically operate in conjunction with adrenergic receptors in the autonomic nervous system control of mammalian smooth muscle.

The following extended account of the model focuses on the state of the subject (dissipative or conservative). Biofeedback can be fatal due to cardiac failure for subjects in an exhausted state, unless attention is given.

In the paper, emphasis is placed on catecholamine stimulation and a temporal pattern of responses is obtained. It has been established that catecholamine secretion is not only of short duration but also persists for long periods (minutes or even hours) [11]. To take account of this, the authors have designed 1^st ^order and 2^nd ^order systems. In the 1^st ^order system the response decays without oscillation during a short catecholamine secretion phase, whereas the 2^nd ^order system represents a prolonged period marked with oscillation, concomitant with adrenergic stimulation leading to vasoconstriction and vasodilatation.

**Figure 2:**
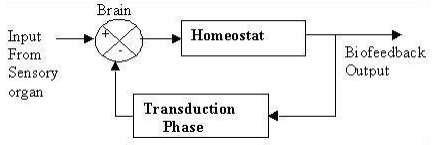
Biofeedback Circuit.

A comprehensive biofeedback model consists of a brain, homeostat and transduction phase (Fig.2). The sensory organs are responsible for biofeedback stimulation. Biofeedback stimulates the nervous system concomitantly with homeostatic regulation of the body through hormonal activation. The role of the brain is central, adjusting the system in accordance with the biofeedback stimulus received from the sensory organ. Without the brain there would be no output response. Biofeedback stimulates the subconscious part of the brain, and depends upon the nature of stimulus received from the sensory organ in the subject's particular current environment. Both the conscious and subconscious parts of the brain are important in biofeedback. Dreams during sleep are sometimes responsible for locomotor action evoked through stimulation of subconscious parts of the brain.

Here, input stimulus to the biofeedback system is a step function while the homeostatic output response is exponential. The input stimulus may be optical (e.g. flash of light), auditory (e.g. tone), tactile (e.g. a blow to the Achilles tendon), or a direct electrical stimulation of some part of the nervous system.[8] Any sinusoidal or ramp input can be simplified by expressing it as a function of step inputs. For this reason the input is taken as a step. In this particular model, the output responses are of two types: exponential rise and exponential decay. Exponential rise signifies that the system is unable to withstand the biofeedback stimulus, depending on the responses of homeostat. Exponential decay signifies a normal homeostatic response. The homeostatic responses are regulated mainly by the functioning of the kidney and heart in tandem.

**Figure 3:**
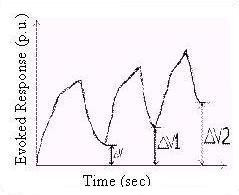
A biofeedback output with multiple responses.

A complex biofeedback output with multiple responses is shown in Fig. 3. ΔV is the residual homeostatic output level. In practice, subsequent biofeedback output responses occur, as shown. The residual homeostatic output level at each stage can sometimes exceed the corresponding value in the previous stage, depending on homeostatic responses.

**Figure 4:**
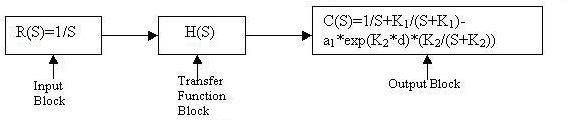
Block diagram representation of biofeedback output with single response.

A generalised GSR model was chosen.[9] For a step input, the body's biofeedback output response is identical to that illustrated in Fig 1. The GSR output was simulated using MATLAB 6.0. Different time constants for the rising and decaying phases were considered for simulation within a fixed interval. Simulation in this model was facilitated by the use of SIMULINK. Knowing that the input is a step and the output exponential, the entire transfer function of the system could be represented by the respective blocks (Fig. 4). K_1 _and K_2 _are the inverse time constants for the rising and decaying phases of the biofeedback output respectively; a_1 _is the peak value of the of the biofeedback output response.

## Methods and Results

**Figure 5:**
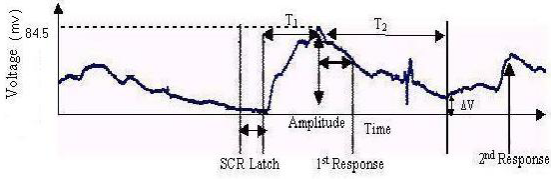
Galvanic skin response of a subject of a particular age.

**Figure 6:**
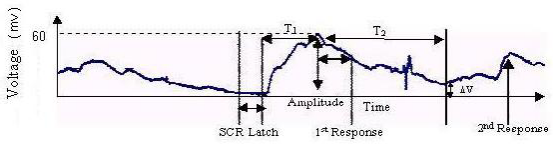
Galvanic skin response of another subject of the same age.

**Figure 7:**
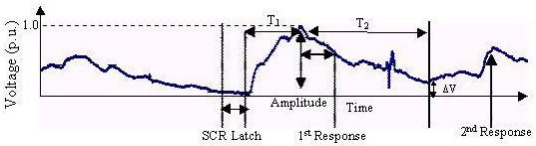
Fitting (per unit values) of data in Fig 5 and Fig 6.

The p.u. (per unit) scale values signify normalisation of the curve to correlate a particular physiological phenomenon such as GSR. Qualitatively similar physiological responses can be fitted by a single curve, irrespective of amplitude, if per unit values are chosen. From Figs. 5, 6, 7 we see that GSRs, qualitatively identical but of different amplitudes, are fitted by the single curve (Fig. 7).

**Figure 8:**
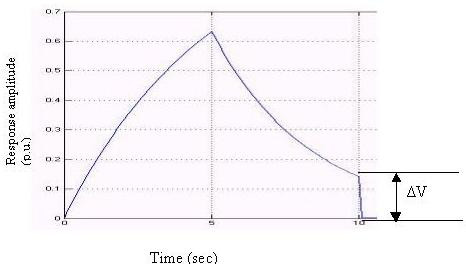
Biofeedback output with single response.

In this model (Fig. 8) the output is a single response. The values of K_1 _and K_2 _are taken as 0.2 and 0.3 and the time periods for the rising and decaying phases are taken as 5s, to correlate with the characteristic GSR response in biofeedback [9].

**Figure 9:**
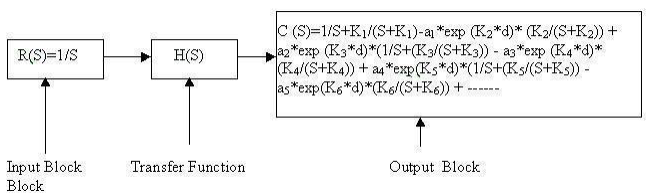
Block diagram representation of biofeedback output with multiple response.

From Fig. 8 the residual homeostatic output level, ΔV, is calculated as 0.142 p.u. Now by keeping K_2 _fixed we can change the value of K_1 _and observe changes in the value of the residual homeostatic output. For i) K_1 _= 0.2, ΔV = 0.1418 p.u; ii) K_1 _= 0.25, ΔV = 0.142 p.u; and iii) K_1 _= 0.15, ΔV = 0.1422 p.u. We can conclude that the residual homeostatic output level does not depend on the time constant of the rising phase of the biofeedback output response. In a real biofeedback system (in this case GSR), there may be more than one response. In that case the entire transfer function can be represented by a block diagram (Fig. 9).

In respect of the homeostatic output level in GSR, the constants a_1, _a_2, _a_3 _relate to the peak value; a_2_, a_4 _represent residual output level. K_1_, K_3_, K_5 _respectively indicate the slopes, i.e. the inverses of the respective time constants of the successive rising phases of the GSR; and K_2_, K_4_, K_6 _respectively represent the inverses of the e time constants of the successive decaying phases. These constants are selected so as to represent the GSR attributable to activation of sweat glands concomitant with stimulation through catecholamine [9,13]. The hormonal stimulation helps elicit physiological responses that obey an exponential law with rising and decaying phases.

### Case-1

**Figure 10:**
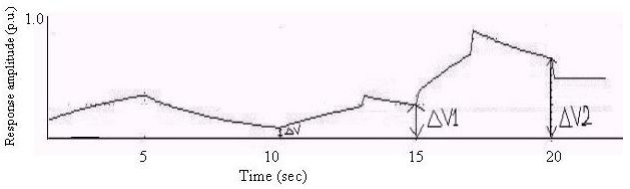
Response vs. Time (Case-1).

In the case of biofeedback with multiple responses, the K_1 _and K_2 _values for successive responses are taken as 0.2 and 0.3 respectively and K_3_, K_5 _and K_4_, K_6 _have values identical to K_1 _and K_2 _(Fig. 10). The time periods for the rising and decaying phases of successive responses are matched separately with the characteristic curve of the GSR response. From Fig. 10 we observe that ΔV increases in successive responses.

### Case-2

**Figure 11:**
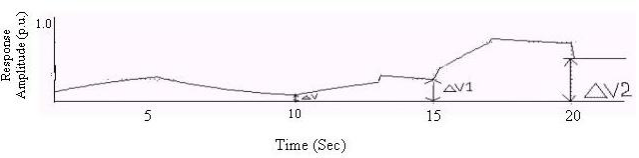
Response vs. Time (Case-2).

Here (Fig. 11) K_1 _= 0.2 and K_2 _= 0.3; K_3 _= 0.1, K_4 _= 0.09; K_5 _= 0.3, K_6 _= 0.5; and the time periods of the 2^nd ^and 3^rd ^responses are taken to be half of the 1^st ^response.

### Case3

**Figure 12:**
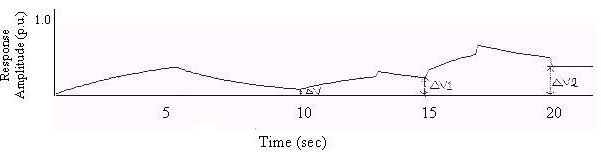
Response vs. Time (Case-3).

Here (Fig. 12) K_1 _= 0.2, K_2 _= 0.3, K_3 _= 0.05, K_4 _= 0.03, K_5 _= 0.02, K_6 _= 0.01; again, the time periods of the 2^nd ^and 3^rd ^responses are taken to be half of the first response.

In all these cases we see that the residual homeostatic output level increases for each successive response [9].

**Figure 13:**
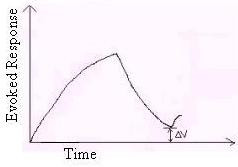
Biofeedback output.

**Figure 14:**
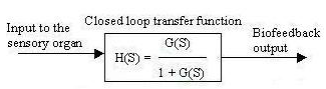
Block diagram representation of closed loop transfer function with unit feedback.

With unity biofeedback the closed loop biofeedback transfer function is given by H(S) = G(S)/(1+G(S)), where G(S) is the open loop transfer function and the biofeedback output is given by Fig. 13. Now the whole system can be shown by a block diagram representation in Fig. 14.

**Figure 15:**
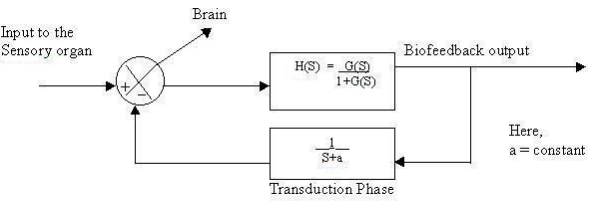
Block diagram representation of system incorporating 1^st ^order transduction phase.

Here the unit feedback control system is converted into an open loop control system, where the closed loop transfer function becomes an open loop transfer function. We next studied the output response when the transduction phase was incorporated into the feedback loop of the biofeedback system. The result can again be shown by a block diagram (Fig. 15). In the first order transduction phase, the constant 'a' represents exponential rise or decay during the phase of catecholamine activation [4-6].

**Figure 16:**
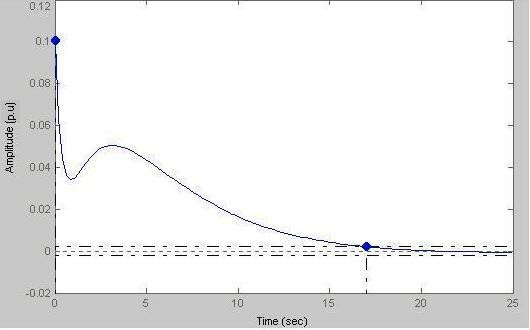
The biofeedback output response when the 1st order transduction phase is incorporated in the feedback loop.

The transduction phase can be either conservative or dissipative. Depending on the nature of the transduction phases, the biofeedback output of a closed loop model as shown in Fig. 16 will typically show the relevant characteristic responses. The expression for dissipative and conservative systems due to incorporation of the transduction phase is:

Tp(Φ_d_) = Φ_d0 _± ∂(ψ_d_)/∂t and Tp(Φc) = Φc_0 _± ∫(ψ_c_)dt

where Φ_d0 _and Φc_0 _are the initial states of the dissipative and conservative system respectively, ψ_d _is the time dependent 1^st ^order dissipative system and ψ_c _is the time dependent 1^st ^order conservative system. Here, the transduction phase signifies the state of the internal environment of the subject [11]. It reflects the topological asymmetry of cellular organization, which shows a relaxation jump associated with hydrophobic linkages among polar heads [1].

Depending on the state of the subject, homeostasis is perturbed in a conservative system. This is the first order system transduction phase where the value of a is taken as 2 and the output appears as

### Case-I

Here peak amplitude = 0.101 p.u and settling time = 17 s

From Fig.16 we see that the exponentially decaying output phase indicates that the subject returns to the original state within a time frame depending on the duration of the catecholamine signal. When the 2nd order transduction phase is incorporated into the biofeedback loop, the block diagram representation of the system is **shown below**.

**Figure 17:**
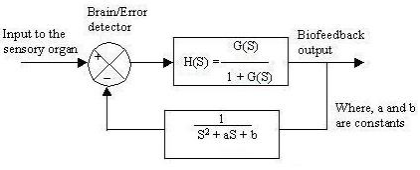
The block diagram representation when the 2^nd ^order transduction phase is incorporated in the feedback loop.

**Figure 18:**
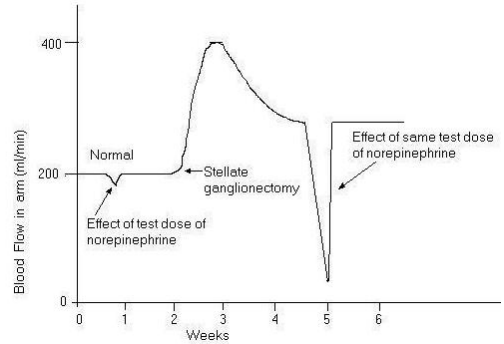
Effect of sympathectomy on blood flow in the arm and the effect of a test dose of norepinephrine before and after sympathectomy (lasting only 1 minute or so), showing *supersensitization *of the vasculature to norepinephrine.

To represent the 2^nd ^order transduction phase, the constants 'a' and 'b' are selected so that there will be simultaneous exponential rise and decay (Fig. 17). This is shown in Fig. 18, which illustrates the catecholamine activation phase for a normal subject (dissipative system) [4,5,11,13]. Fig. 18 represents the transduction of blood flow mediated by catecholamine.

**Figure 19:**
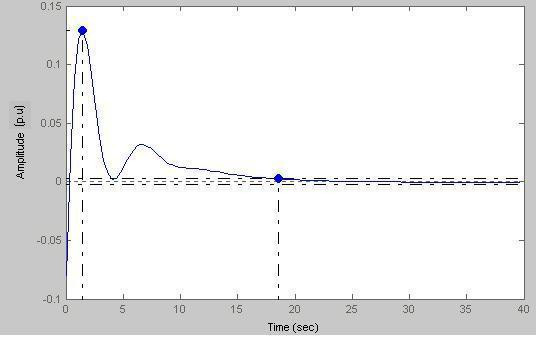
Biofeedback output response when 2^nd ^order transduction phase is incorporated in the feedback loop.

Assuming a = 1, b = 1 we can have the system response in Fig. 19.

### Case-II

Here peak amplitude = 0.129 p.u and settling time = 19 s. Fig. 18 illustrates the fluctuations of parameters such as blood pressure and pulse rate, which persist for a certain period of time concomitant with the sustained catecholamine signal.

Keeping the value of b fixed at 1 and by putting a = 0.5 we obtain the output response shown in Fig. 19.

### Case-III

**Figure 20:**
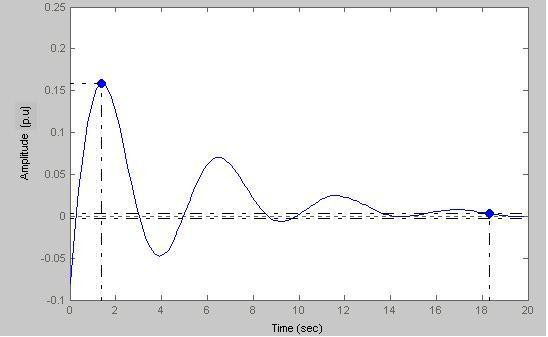
Response amplitude vs Time (a = 0.5).

Here (Fig. 20) peak amplitude = 0.158 p.u and settling time = 18.3 s

### Case-IV

**Figure 21:**
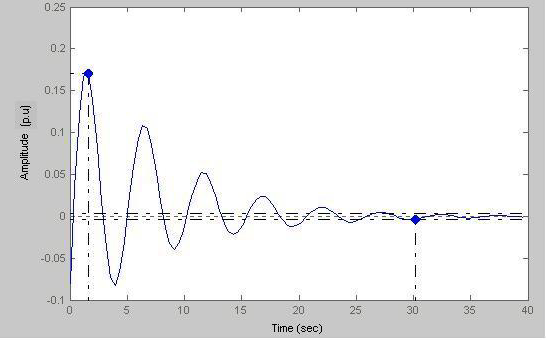
Response amplitude vs Time (a = 0.3).

Here (Fig. 21) peak amplitude = 0.171 p.u and settling time = 30.2 s

### Case-V

**Figure 22:**
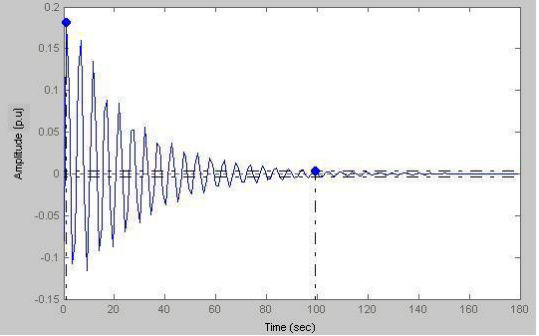
Response amplitude vs Time (a = 0.1).

Here (Fig. 22) peak amplitude = 0.181 p.u. and settling time = 99.2 s

Figs. 19, 20, 21, 22 model states with different values of 'a'. With decreasing 'a' values, the settling time increases with the increase of oscillations. This is true for a subject with sustained biofeedback.

### Case-VI

**Figure 23:**
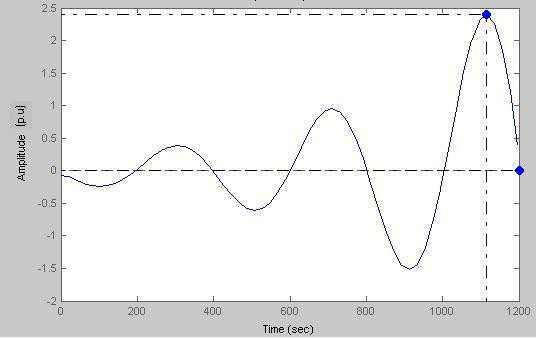
Response amplitude vs Time (a = 0.015).

Peak amplitude = 2.41 p.u and damping freq = 0.002463Hz (Fig. 23).

### Case-VII

**Figure 24:**
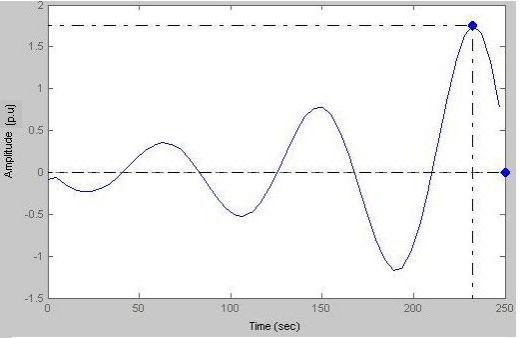
Response vs Time (when damping is absent, i.e. a = 0).

Here peak amplitude = 1.76 p.u and damped frequency = 1/(126-40.7) = 1/85.3 = 0.01172Hz (Fig. 24).

Figs. 23, 24 represent a subject with a permanent disorder; the biofeedback stimuli cause the disorder to be manifest. By putting a = 0 we can have the output response. Here we clearly see that sustained oscillations amplify in a conservative transduction phase due to the prolonged period of catecholamine activation.

## Conclusion

The features of both dissipative and conservative systems are represented in this comprehensive model, which is based on catecholamine activation. The transduction phase of the 2^nd ^order system in biofeedback can act as either a dissipative or a conservative system depending on the system dissipation factor (which is related to catecholamine production). For a dissipative system the catecholamine signal is of shorter duration, whereas for a conservative system it survives for a longer period. Biofeedback can sometimes produce complex responses in biological systems depending on how sustained the catecholamine signal is; these complexities are represented by the present model. In the context of this paper, the envelopes of the exponentially rising and decaying phases also represent the stimulation of adrenergic receptors in monotonic phase concomitant with the catecholamine production. Adrenergic and cholinergic receptors have opposing roles in the autonomic nervous system. Downregulation of sympathetic innervation via adrenergic receptor is followed by enhancement of the cholinergic receptors involved in parasympathetic stimulation in smooth muscle. Conversely, noradrenergic enhancement is diminished as cholinergic neurotransmission becomes established. Thus it may be concluded that cholinergic receptors automatically participate, along with adrenergic receptors, in the autonomic nervous system control of mammalian smooth muscle function.

In this paper a new conceptual approach has been taken to modeling dynamic responses in biofeedback that depend on hormone activity, by introducing homeostats and transduction phases in the feedback path.

## Competing Interests

As head of the Department of Electrical Engineering, Jadavpur University, Professor Basak requested the University authorities to obtain membership of  and the university has given due consideration to this request.

## Authors' contributions

Professor T. K. Basak received a third world scientist award from ICTP, Trieste, Italy and worked with Professor A. Glilozzi in the Dept of Biophysics, University of Genoa, Italy in 1985. He furnished the innovative idea in the present paper and provided comprehensive guidance to the team from the outset. After completing his Masters degree in electrical engineering under the supervision of Professor Basak, Mr. Suman Halder began Ph.D. work under the same supervisor and was involved with the work until the completion of the paper. Ms. Madona Kumar and Mrs. Renu Sharma were Masters students under Professor Basak's supervision and participated in the completion of the work and the preparation of the manuscript. Ms. Bijoylaxmi Midya' a lecturer in the Department of Applied Electronics & Instrumentation Engineering, Haldia Institute of Technology, Haldia, is doing Ph.D. work under Prof. Basak and contributed to the completion of the paper.

## References

[B1] Gliozzi A, Bruno S, Basak TK, Rosa MD, Gambacorta A (1986). Organization and Dynamics of Bipolar Lipids from Sulfobus Solfataricus in Bulk Phases and in Monolayer Membranes. System Appl Microbiol.

[B2] Basak TK, Aich NS (1990). Photo induction on fish-anabas testudineus,. Proceedings of IEEE EMBS International Conference.

[B3] Basak TK (1991). Entropy transduction on photoinduction,. Proceedings of IEEE EMBS International Conference.

[B4] Basak TK (1992). Catecholamine interaction in blood pressure transduction,. conference on Medimechatronics, Malaga, Spain organized by Bristol University UK.

[B5] Basak TK, Islam R (1993). Role of sensory hormones in estimation of blood pressure transduction,. Proceedings of IEEE EMBS International Conference, 0-7803-1377.

[B6] Basak TK, Dutta J (1994). pH dependence of the interactions in blood pressure transduction,. proceeding of IEEE EMBS International conference, 0-7803-2050.

[B7] Basak TK (1996). pH dependent transduction in renal function regulation,. proceeding of 18th Annual International conference of the IEEE EMBS, no 0-7803-3811 Amsterdam.

[B8] Basak TK, Dutta JC, Ghosh SK (2001). Some optical characteristics of biomembrane in the development of biosensors,. International society for optical engineering SPIE, USA.

[B9] Liew SP (2001). Monitoring galvanic skin responses in functional magnetic resonance imaging. Ph D thesis.

[B10] Tarvainen M, Koistinen A, Valkonen-Korhonen M, Partanen J, Karjalainen P Principal component analysis of galvanic skin responses. IEEE Trans Biomed Eng.

[B11] Guyton, Hall (2003). Textbook of Medical Physiology: Elsevier.

[B12] Wintrobe, Thorn, Adams, Braunwald, Isselbacher, Petersdorf (1972). Principles of Internal Medicine.

[B13] Tian H, Habecker B, Guidry G, Gurtan A, Rios M, Roffler-Tarlov S, Landis SC (2000). Catecholamines Are Required for the Acquisition of Secretory Responsiveness by Sweat Glands,. J Neurosci.

[B14] Krizsan-Agbas D, Zhang R, Marzban F, Smith PG (1998). Presynaptic adrenergic facilitation of parasympathetic neurotransmission in sympathectomized rat smooth muscle. J Physiol.

